# The Virginia Medical and Surgical Journal

**Published:** 1853-04

**Authors:** 


THE VIRGINIA MEDICAL AND SURGICAL JOURNAL.

We have received the first number of this Journal, and hasten to express the favorable opinion with which we have risen from its perusal. We have no hesitation in saying, that if the editors are able to keep up to the high mark which they have attained in their first issue, their Journal will take a high rank among the medical serials of the day. It is printed on good paper and type, and its matter is of corresponding excellence. We should hope, that among the three thousand physicians of Virginia, it might find, even amid all the pressure of our medical journalism, an adequate body of contributors and subscribers; but whether the profession of the "Old Dominion' rally around it as they ought, or not, we are very sure the enterprising editors have not embarked in "a twelve months voyage to obscurity and the chaos of failures." Every page of their work bears the impress of industry and care, and such diligence, backed by such qualifications for conducting a journal of medicine, will not long be overlooked by the profession. We welcome it cordially, and trust that it may have a long career of usefulness and popularity.

The Virginia Medical and Surgical Journal is published at Richmond, and is to appear monthly. Its editors are George A. Otis, M.D., and Howell L. Thomas, M.D.

